# A Qualitative Analysis of Impact of TRIANGLE Digital Platform Based Forum Intervention on Children and Young People with Anorexia Nervosa and Their Carers

**DOI:** 10.1192/bjo.2026.11082

**Published:** 2026-06-30

**Authors:** Gabriella Costain, Ashish Kumar, Hubertus Himmerich, Janet Treasure

**Affiliations:** 1 Mersey Care NHS Foundation Trust, Liverpool, United Kingdom; 2 Kings College, London, United Kingdom

## Abstract

**Aims:**

To explore how young people with a diagnosis of Anorexia Nervosa and their carers describe shifts in understanding, thinking and perspective during a clinician-moderated online forum group within a children’s eating disorder service.

**Methods:**

Digital interventions are increasingly used within eating disorder services to provide peer connection, psychoeducation and clinician support. Young people with a diagnosis of Anorexia Nervosa and their carers from Mid Mersey children’s eating disorder service were offered digital platform based clinician moderated forum groups as part of TRIANGLE research project, which generated discussions around their illness and their understanding and perceptions of recovery from Anorexia Nervosa. Previous qualitative studies have shown perspectives and sense making inAnorexia Nervosaare central to recovery processes and online support groups can reduce isolation and influence emotional regulation, however, little is known about how shifts in understanding, meaning and perspectives of an eating disorder are experienced within online group intervention in both patients and their carers. This study addresses that gap by analysing group transcripts to identify the processes through which participation may reshape cognitive and emotional narratives related to eating disorder experience and recovery.

Verbatim transcripts from three cohorts of seven consecutive online forum groups were analysed using reflexive thematic analysis, using NVivo software the discussions of 13 youngpeople with a diagnosis of Anorexia Nervosa or Atypical Anorexia Nervosa and 13 of their carers were analysed with attention to changes in participants’cognitive and emotional narratives over time.

Ethical considerations: This included informed consent for research use, anonymisation of transcripts and processes for escalation of safeguarding concerns within the groups. The ethical approval for the TRIANGLE study was taken from REC Liverpool, REC Ref – 25/NW/0029.

**Results:**

Preliminary patterns suggest that young people experience changes in understanding and insight into the impacts of eating disorders, greater capacity for emotional awareness and reframing of ED identity over the course of the online groups. Carers also experience shifts in understanding of young people’s eating disorder behaviours but also a greater ability to reflect and an increased perspective of the impact of their own emotional regulation. Clinician support provided prompts to reframe experiences and promote self-reflection, increased awareness about concepts such as expressed emotion and accommodation within the participants.

**Conclusion:**

These findings suggest that digital group interventions within children’s eating disorder services can support recovery by facilitating shifts in cognitive, emotional and meaning based processes that may underpin change and motivation for recovery.

